# Radium in Deafness: Hyoscine Injections and a Captious Critic

**Published:** 1914-05-02

**Authors:** 


### RADIUM IN DEAFNESS.

### Hyoscine Injections and a Captious Critic.

In the issue of the *Medical Press*, dated April 15, 1914, the Editor saw fit to print a letter which he had received from a member of the medical profession criticising in turn The Hospital, some doctors at Preston, and the whole surgical profession. Inasmuch as certain personal considerations, of which the Editor of the *Medical Press* was doubtless uninformed, seem possibly to have inspired this wholesale denunciation, it is due both to that paper and to ourselves to discuss briefly the whole incident.

On March 21 there appeared in a Special Number of The Hospital an article on radium, specially contributed by a correspondent who had taken great pains to present an accurate synopsis of the existing state of expert medical opinion concerning the advantages and disadvantages of this substance in therapeutics. The Special Number was distributed to the whole of the medical profession; and the article on radium, which formed much the largest individual section of the issue, must have been read by many thousands of doctors. Only one paragraph in it elicited any expression of dissent or disapproval, and that from only a single writer. The statement to which exception was thus taken was very brief, and was to the effect that in the treatment of deafness radium has proved disappointing. To this statement Dr. H. D. McCulloch demurred in a letter which was printed in our correspondence columns (The Hospital, March 28, 1914); from it were omitted certain offensively worded paragraphs which did not in any way affect the writer's argument, and to it a brief note was appended editorially, giving the reference in the specialists' journal upon which our contributor's paragraph was based. Not only did we thus place our space at Dr. McCulloch's disposal; we further afforded hospitality to another letter later on (The Hospital, April 18, 1914), in which a doctor testified to the fact that a relative had improved in hearing while under Dr. McCulloch's care. It will be seen that no lack of courtesy can reasonably be alleged against the Editor of The Hospital so far as his dealings with this doctor are concerned.

In The Hospital of April 4 (p. 7) a very short account was given of a fatality at Preston following the use of radium. A tube of this metal had been inserted into a patient's nostril, whence it had been swallowed accidentally. Emetics having failed, an operation was performed for the recovery of the radium. As part of the process of anæsthesia an injection of atropine and hyoscine was given, and to the latter drug was attributed the death of the patient later on by those who gave evidence at the subsequent inquest. The only comment offered in The Hospital on this sad story was, in substance, that more care should be exercised in future to prevent such accidents as the swallowing of this radium tube. This comment had no connection whatever with the article on radium treatment of March 21; nor was it by the same correspondent; nor did it bear in any way upon the letter which we had received from Dr. McCulloch. We see nothing to modify or to withdraw in it.

In some way or another this harmless paragraph seems to have annoyed Dr. McCulloch. We should have been tempted to attribute his subsequent actions to personal spleen had we not been so entirely innocent of any intent to cause offence to him or of any action that could be so construed. At all events, he composed a letter bristling with criticism, based upon the paragraph in question, and sent it to the *Medical Press*, in which it was printed. This letter is so remarkable that it deserves a short analysis. It begins by criticising The Hospital for suggesting that tubes of radium inserted in canals such as the nose should be securely fastened so that they cannot escape; and almost in the same breath proceeds to criticise the medical men in charge of the case for failing to do so. After this striking inconsistency, the letter pronounces that a panic seized upon the patient's medical attendants. These gentlemen are no doubt quite capable of defending themselves against this accusation; but it should be added that Dr. McCulloch's suggestions of what should have been done strike us as peculiarly useless and as involving a most dangerous delay.

He then proceeds to denounce the use of hyoscine preliminary to anæsthesia for the operation. It may be that the majority of the medical profession will some day adopt the view that the risks of this drug outweigh its advantages in such circumstances. But at the moment it is widely used, and by some of the leading experts; so that it is not fair to infer culpable stupidity on the part of an individual country practitioner who uses it in ordinary dosage (as in this case). Diverging from this topic, Dr. McCulloch vehemently attacks surgeons in general for their alleged frequent mistakes in diagnosis, whereby laparotomies are commonly performed for supposed perforated gastric ulcer or gall-stones when no such condition exists. We leave it to any competent surgeon to "place" Dr. McCulloch, on the strength of this paragraph; and we wonder that it did not occur to the Editor of the *Medical Press* to do likewise.

And now we have done with this disgruntled gentleman. We do not know, nor do we ask, why he went out of his way to attack The Hospital, since we had already given him ample ventilation of his fancied grievance against our contributor on radium therapy. But we do think the Editor of the *Medical Press* owes himself and us some explanation of his action in permitting Dr. McCulloch's letter to appear in that paper. We are content to leave it at that and to the judgment of our readers, assuring them that in what precedes this sentence nothing but the simple facts of the case have been set down, without concealment, distortion, or exaggeration.

